# Leading with Integrity: Impact of Ethical Leadership on Performance of Healthcare Professionals in Saudi Arabia

**DOI:** 10.3390/healthcare13243205

**Published:** 2025-12-08

**Authors:** Badr K. Aldhmadi, Rakesh Kumar, Bilesha Perera, Mohammad A. Algarni

**Affiliations:** 1 Department of Health Management, College of Public Health and Health Informatics, University of Ha’il, P.O. Box 2440, Ha’il 55476, Saudi Arabia; b.aldhmadi@uoh.edu.sa (B.K.A.); bileshap@med.ruh.ac.lk (B.P.); 2 Faculty of Medicine, University of Ruhuna, Galle 80000, Sri Lanka; 3 Faculty of Economic and Administration, King Abdulaziz University, Jeddah 21589, Saudi Arabia; maaalqarni2@kau.edu.sa

**Keywords:** ethical leadership, employee performance, organizational support, healthcare, Saudi Arabia

## Abstract

**Background/Objectives:** Ethical leadership (EL) propels and enhances employee performance (EP), especially in healthcare, where ethics are paramount. However, existing research lacks a focus on how EL functions within Saudi Arabia (SA)’s public healthcare context. Primarily, this research investigates how EL directly affects EP. The research also investigates how organizational support (OS) influences EP and moderates the EL-to-EP relationship. **Methods**: This cross-sectional study consisted of 312 responses from doctors, nurses, and other administrators within Saudi public healthcare units. To analyze the collected data statistically, structural equation modeling (SEM) was opted for with the help of Smart-PLS 4. It helped to assess the direct effects of EL and OS on EP and further examine OS’s moderating role. A multigroup analysis (MGA) was also conducted in comparative form. It examined subgroup variations across gender, age, marital status, experience, and departmental affiliation. **Results**: The findings confirmed a positive impact of EL on EP. Moreover, a positive effect of OS on EP was also confirmed. Similarly, OS strengthened the positive effect of EL on EP. The MGA revealed variations across employee groups. It offered practical insights into how EL and OS function in diverse organizational contexts. These differences across groups reflect cultural and structural features of Saudi public healthcare. **Conclusions**: The extended research contributes to the literature on ethical leadership (EL) theory by applying contextual and demographic contingencies within the Saudi public healthcare sector. It also introduces OS as a significant moderator and provides actionable implications for improving performance through context-sensitive leadership and support strategies.

## 1. Introduction

The healthcare sector is the cornerstone of national development, ensuring the well-being and productivity of the population. This sector has garnered significant attention and investment under the Vision 2030 initiative in SA. It aims to transform the country into a leading healthcare provider at both the regional and global level [1]. As the industry continues to shift, the complexities of healthcare environments require effective and, most importantly, ethical leadership. Trevino et al. described that ethical leaders are distinguished by their behavior, such as honesty, fairness, integrity, and concern for others [2]. Hence, in healthcare settings, such leadership is especially needed to shape staff behavior, strengthen trust, and support high-quality clinical and administrative performance.

In a sector with high stakes, leaders’ actions directly determine patient care along with staff morale, as well as overall organizational success. Ethical leaders establish a moral tone and substitute an environment of trust and reciprocated respect, which is crucial in high-stress healthcare settings [3]. In SA, where organizational behavior is heavily influenced by cultural and religious values, EL has added significance [4]. Islamic principles, deeply embedded in Saudi society, emphasize justice, fairness, and integrity [5]. These qualities are intrinsic to EL.

In the SA context, EL is theoretically and practically influential in increasing EP, satisfaction, and commitment [6,7,8]. Surprisingly, there is little research around SA in healthcare. The OS may enhance or dampen these effects, but its role is under-investigated. Comparisons across subgroups by gender, age, marital status, tenure, and departments are limited; therefore, practical implications are scarce. Filling these gaps is essential in enhancing EP and supporting Vision 2030.

According to Koleva [9], EL is central to the enablement of EP in the Middle East; however, deeper analysis will be required to understand its impact within SA healthcare. Unique cultural, organizational, and structural contexts of the sector may shape EL perceptions and practices [4]. SA demonstrates unique cultural and organizational characteristics. These include high power distance [10] and collectivist norms that emphasize loyalty to in-groups and family ties [11]. In addition, tight, high-context communication patterns shape how workplace support and leadership messages are interpreted [12]. These can strongly shape how EL and OS are perceived and enacted, in contrast to Western or broader Middle Eastern contexts. Such contextual differences provide a clear rationale for why Saudi healthcare outcomes may differ from prior studies. The potential moderating role of OS on EP in these contexts is not well explained, to which this study responds with an MGA illustrating the subgroup characteristics influencing the EL–EP link. Understanding such dynamics is important for articulating correct leadership strategies that are both culturally appropriate and effective in ensuring high performance within the healthcare sector.

The primary aim of this research is to investigate how EL affects EP in the public healthcare setting of SA. It also investigates OS’s moderating effect on EL in relation to its impact on EP along with task performance (TP), contextual performance (CP), and adaptive performance (AP). Therefore, this study has the following specific objectives:

1. To analyze the behavior of EL in determining EP and its subdimensions (TP, CP, and AP) in the public healthcare sector of SA.
2. To analyze the influence of OS on EP within SA’s public healthcare sector.
3. To determine the moderating role of OS for EL and EP (along with TP, CP, and AP) relationships as per their defined context.
4. To compare these effects across demographic and organizational groups.

This research significantly contributes to leadership in terms of theory and practice. It also advances organizational behavior studies, focusing on the healthcare sector in SA. First, it extends the existing EL literature by investigating its effects in a non-Western and culturally different context, thereby adding value from a global perspective. This study identifies OS as a moderator in the influence of EL on EP and investigates subgroup variations empirically to gain a detailed insight into leadership dynamics. In the healthcare context, where EP has a direct effect on the care given to patients, findings recommend culturally and religiously appropriate leadership for better EP and OE in the sector.

## 2. Theoretical Review

EL is commonly defined as the demonstration of appropriate, norm-based conduct through personal actions and interpersonal relationships, combined with the active promotion of such conduct among followers. The theoretical basis of this research is the EL theory proposed by Brown et al. [13]. This theory states that leaders should behave ethically. According to Ete et al. [14], leaders influence employees by role-modeling appropriate behavior, setting clear standards of ethics, and building a trusting environment of integrity. EL is perfect for applications in sectors such as healthcare [15]. In this sector, the stakes are high, and the consequences of unethical behavior can be considered high, directly affecting patient care and organizational reputation. According to EL theory, leaders are both moral persons characterized by fairness, honesty, integrity, and concern for others, and moral managers [2]. These characteristics cultivate ethical conduct through communicative, rewarding, and accountability structures. Based on Social Learning (SL) theory, employees learn through observing credible role models [13]. In healthcare, where the work is highly interdependent and prone to errors, ethical leaders reinforce norms, enhancing psychological safety. Furthermore, they build an ethical climate that sustains responsible decisions and professional conduct.

According to Kleshinski et al. [16], when leaders consistently behave in an ethical manner, they set a model that encourages all employees to do so, too. This enhances EP because individuals feel morally comfortable in the company and agree on the values worth maintaining. Not every group is equally influenced; for instance, ethical modeling may be more effective in younger or junior employees than in senior staff—a reason why comparing different groups has been underlined [17]. The current study also attempts to determine the moderating role of OS in the association between EL and EP. According to OS theory as proposed by Eisenberger et al. [18], workers who perceive more OS feel valued and thus are more committed and better performers. OS might enhance EL effects by building trust in leadership, but that varies across groups—clinical and support staff. This is important as employees experience daily stress and burnout [19]. Perceived OS develops a psychologically safe environment, where employees feel safe reporting concerns or seeking help. In high-pressure healthcare, such support reduces stress and burnout and encourages a greater willingness to perform at higher levels. Therefore, POS enhances the EL–EP relationship by reinforcing perceptions of trust, fairness, and available resources.

Social exchange (SE) theory as proposed by Blau [20] provides additional insights. The more employees perceive support from the organization, the better they will perform, reinforcing both a supportive atmosphere and the relationship between leader–member exchange and perceived performance. [21]. This is a reciprocal mechanism that varies with demographics and organizational traits; hence, subgroup analysis becomes quite crucial in capturing nuances. From the SE perspective, EL would signal fairness and respect and thus ensure positive attitudes and behaviors of employees. Coupled with perceptions of OS, this becomes a reinforcing cycle of trust, obligation, and motivation that enhances both TP and CP.

EL theory directly relates leadership to performance in healthcare, whereas OS theory reveals how perceived support enhances such outcomes, and SE theory explains the motivational and reciprocal employee reactions. Collectively, they predict the overall leadership–performance links and subgroup variability. Integrating them provides a base to examine the direct and moderating EL and OS effects and explains subgroup differences through multigroup analysis. This study applies EL theory to a non-Western healthcare context, extending prior, Western-focused work. It shows that the underlying EL mechanisms are not universal but shaped by socio-cultural and institutional dynamics in non-Western settings. This theoretical integration is particularly relevant in healthcare systems where there are hierarchical structures, role interdependence, and high-stakes decision-making. It amplifies the importance of ethical and supportive leadership.

## 3. Literature Review and Hypothesis

The literature review examines how EL and OS impact EP and how OS moderates this relationship. In countries like SA, for example, with unique contexts regarding healthcare, EL tends to increase the performance of EP; however, effectiveness depends on the OS of those policies related to it. Recent studies confirm that such effects differ according to demographic and organizational groups, and hence further comparative study is required. The next sections discuss these associations and present hypotheses.

### 3.1. Ethical Leadership and Employee Performance

EL has been studied across industries, where leaders who model ethical behavior seem to positively affect employee outcomes. EL builds an atmosphere of trust, fairness, and moral integrity, which is important for improving EP [22,23,24]. Consequently, the followers of ethical leaders are expected to participate in ethical behaviors [25]. These employees are highly satisfied with their jobs and committed to organizational goals. This is particularly significant in healthcare, as the ethical performance of leaders directly affects the delivery of quality care provided to patients. In practical terms, EL enhances healthcare delivery by improving multidisciplinary teamwork. It clarifies handovers, increasing adherence to patient safety protocols, and promoting consistent ethical decision-making under pressure. These pathways show how ethical leaders influence staff behavior not only through value alignment but also via tangible gains in care coordination and service quality. It also determines the aggregate output of healthcare units [26,27]. However, not all studies have reached conclusions on the size of this effect. Some studies have suggested a conditional effect of EL on performance [27,28]. Moreover, younger or junior employees may be more receptive to EL than senior employees [17]. Moreover, gender and departmental roles may also influence responsiveness [29]. In SA, cultural and religious values further shape these dynamics, reinforcing the importance of ethical norms [30,31]. Therefore, the trends in most studies may support the positive effects of EL on EP. Therefore, based on the literature review, the first hypothesis as well as its sub-hypotheses are as follows:

**H_1_.**  *EL should have a positive impact on EP*.

**H_1a_.**  *EL should have a positive impact on TP*.

**H_1b_.**  *EL should have a positive impact on CP*.

**H_1c_.**  *EL should have a positive impact on AP*.

### 3.2. Organizational Support and Employee Performance

The OS theory asserts that employees with perceived OS are expected to enhance their job output [18]. Healthcare employees, in most instances, face some of the highest stress and burnout levels in any profession [32]. Therefore, OS in such environments has become an increasingly vital factor affecting employee morale and performance. Furthermore, employees’ OS increases their motivation to perform beyond formal job requirements, leading to increased overall organizational effectiveness [33,34]. However, not all studies agree on the effect of OS on EP. This system may be less effective if other determinants such as leadership style or job design do not influence employees’ desires. These factors are crucial for engaging employees in their jobs [35,36]. In SA’s healthcare context, OS would either undermine or improve EP, considering the high level of hierarchy and cultural expectations that are characteristic of the region [37]. Evidence indicates that the effect of OS may vary across groups, including clinical and support staff [38]. These variations arise from differences in workload and role expectations. Thus, the second hypothesis was formulated as follows:

**H_2_.** *OS should have a positive impact on EP.*

### 3.3. Moderating Role of Organizational Support

The influence of EL on performance is less direct, and OS can play a significant moderating role in such instances. A number of studies have already pointed out that the success of EL itself may depend on employees perceived OS [39,40]. Therefore, EL drives a stronger positive effect when workers perceive strong OS. Additionally, a sense of OS creates fertile psychological ground on which heightened feelings of identification and commitment can occur among workers [41]. This increases the positive effect of EL. However, in the absence of adequate OS, EL cannot succeed effectively. Employees may perceive EL as being inconsistent with the broader organizational environment and hence may become disengaged or feel cynical [42]. Moving specifically to the culturally sensitive setting of the SA healthcare sector, organizational dynamics are complex and multifaceted [1]. Moreover, the moderating effect may vary across groups. For example, OS may strengthen the EL–EP link more strongly among junior or clinical employees than among senior or support employees [43]. Based on this reasoning, the following hypothesis and sub-hypotheses are proposed:

**H_3_.**  *OS strengthens the impact of EL on EP*.

**H_3a_.**  *OS strengthens the impact of EL on TP*.

**H_3b_.**  *OS strengthens the impact of EL on CP*.

**H_3c_.**  *OS strengthens the impact of EL on AP*.

### 3.4. Group Differences

Prior research confirms the significance of EL and OS. However, studies also demonstrate that their effects are not uniform across employees [44]. Demographic variables such as gender, age, and marital status influence how individuals perceive and respond to leadership and support structures [45]. Furthermore, professional characteristics, including work experience and departmental affiliation, contribute to shaping these responses in meaningful ways [46]. MGA integration offers a more rigorous way of considering these differences and yields significant theoretical and practical implications [47]. It allows for testing to see if leadership and support have consistent or divergent effects across various demographic and organizational groups. Therefore, in light of this complexity and variability, the study at hand develops a hypothesis.

**H_4a_.**  *The effect of EL on EP differs across employee groups based on gender, age, marital status, work experience, and departmental type*.

**H_4b_.**  *The effect of OS on EP differs across employee groups based on gender, age, marital status, work experience, and departmental type*.

**H_4c_.**  *The moderating effect of OS on the relationship between EL and EP differs across employee groups based on gender, age, marital status, work experience, and departmental type*.

**Figure 1:**
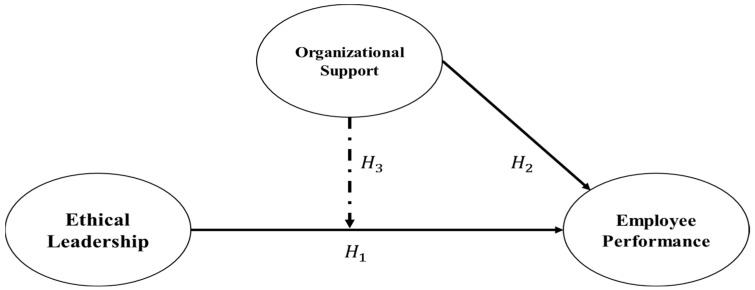
Conceptual model 1.

Figure 1 indicates the conceptual research model 1 for this study based on the objectives, the literature review hypotheses, and the relevant EL theory. H_1_ represents the direct impact of EL on EP. H_2_ indicates a direct effect of OS on EP. This is further moderated by OS based on H_3_ that connects EL and EP.

**Figure 2:**
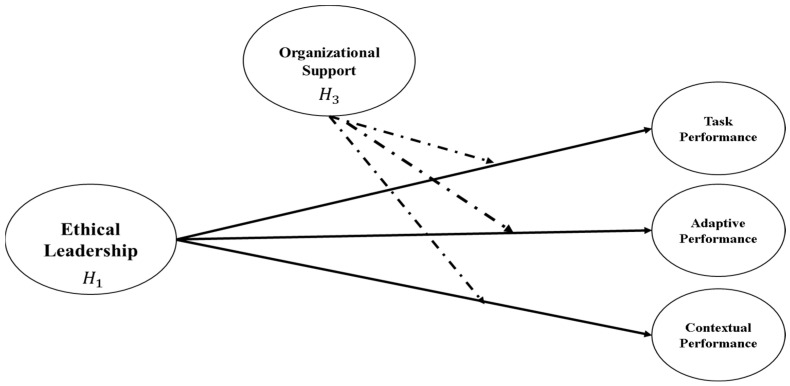
Conceptual model 2.

Figure 2 indicates similar impacts using the sub-hypothesis of H_1_ and H_3_. It includes the direct impact of EL on EP’s three dimensions (TP, CP, and AP) as well as the moderating impact of OS.

## 4. Research Methods

This study implemented a research design which was quantitative by nature. The researchers collected primary data using a structured questionnaire by surveying target respondents. A quantitative approach tested the hypotheses and examined cause-and-effect relationships using numerical data [48]. It used survey responses to prove points or arguments using facts and figures. Deductive reasoning was used in research from pre-existing theories on EL and OS, from which specific hypotheses were developed for empirical testing. The hypotheses were then empirically tested in the SA public healthcare sector. Thus, the general premise was that EL influenced EP, leading to specific conclusions once the data were analyzed to confirm or refine the developed hypothesis. Therefore, this study exemplified a deductive approach in which pre-existing theoretical assumptions were verified through a focused investigation [49].

The study setting was the Hail Health Cluster, SA. It consisted of public-sector health facilities comprising general hospitals, specialized secondary hospitals, and primary care centers. All these institutes had shared centralized governance with hierarchical administration, as is common with most Saudi public healthcare. Public facilities are usually more hierarchical, with strict role boundaries, and decision-making is made more centrally compared to private hospitals. These structural features influence how staff perceive leadership behaviors and OS. The sample spanned doctors, nurses, and administrators from these different SA public healthcare departments, chosen because of the importance of their roles in national health services. Included areas were as follows: cardiology, emergency/critical care, medical imaging, general services, and support units—a broad variety in function. This made subgroup comparisons by gender, age, marital status, experience, and department possible, which was essential in the MGA later in this study. Each department had unique problems and cultures affecting the perceptions of EL and its impact on EP. The final sample was skewed toward cardiology and general healthcare staff due to greater availability, mirroring typical Saudi hospital workforce patterns. In order to counter such biases, MGA-based subgroup analyses were conducted to ensure that findings were consistent among these departments.

The data collection instrument for the targeted participants of this research included a self-administered, close-ended survey questionnaire. It consisted of three parts. Part 1 indicated the purpose of this study, including the exclusive rights of the participants and some prerequisites. Part 2 included questions on respondents’ personal attributes. These attributes included gender, marital status, age, education level, nationality, specialization, work experience, and departmental affiliation within healthcare units in SA. Part 3 concerned the three constructs of the study: EL, OS, and EP (TP, CP, and AP).

The constructs were measured using established scales with proven reliability. EL was the first exogenous construct of this study. It was measured using five items adapted from Brown and Treviño [2]. OS was the second exogenous construct, which was also a moderating variable in this study. It was measured using six items adopted from Suazo and Turnley [50]. In the present study, the endogenous construct was EP. It was measured across three dimensions: TP (four items), CP (seven items), and AP (four items). These dimensions were adopted from Pradhan and Jena [51]. The items and statements attributed to the constructs were measured using a Likert scale. The scale ranged from 1 (“strongly disagree”) to 7 (“strongly agree”). These scales are widely used in organizational research due to their reliability and validity. It makes them well suited for this study’s context of SA’s public healthcare sector.

A pilot test was conducted with a small group of 30 participants in order to ensure the reliability of constructs and their statements. Using the feedback from the pilot testing, the questions/statements were updated. These changes were aimed at enhancing the clarity and improving the overall flow of the questionnaire. The instrument was reviewed by a panel of experts in the fields of leadership, organizational behavior, and healthcare management for content validity. Their comments were then integrated into this final survey. Cronbach’s alpha showed that all constructs had acceptable internal consistency. Moreover, Harman’s single-factor test was also used to identify the potential common method bias (CMB). The results showed that no single factor explained most of the variance. The test result indicated an absence of CMB. The researcher also ensured that ethical standards were adhered to throughout the study. Ethical approval was obtained from the institutional ethics research board of the University of Hail, SA. Target participants were selected using a probability-based simple random sampling method, and their participation consisted of voluntary responses to the survey.

The minimum sample for this study was based on Item Response Theory (IRT) as per Reeve [52]. This suggests that a reasonable sample size may be determined by multiplying the number of items constituting a questionnaire by ten. There were 26 items for the three constructs used in this study. Therefore, a reasonable sample size per IRT was 260 (26 × 10). However, the final number of responses needed to be greater than 260 to control for the probability of missing values or incomplete information. The researcher shared the questionnaire with 360 participants to obtain volunteer responses regarding this study. Out of these, 312 returned completed responses, indicating a very robust response rate of 87%. Missing data were handled carefully: cases with substantial incomplete responses were excluded, while minor missing values were treated using mean substitution. The rate of missing data was minimal, and thus did not affect the robustness of the analysis. To check for non-response bias, early and late respondents were compared on key demographic and construct variables. No significant differences were observed.

Data analysis was carried out using SPSS 27 and Smart PLS 4 for SEM (partial least squares). It estimated participant characteristics in SPSS 27; the remaining analysis, including the measurement model assessment, used Smart PLS 4. Analyses included convergent and discriminant validity, model fit, explanatory power, and SEM. Multicollinearity was checked via VIF, assessing the normality of data for skewness/kurtosis. The results indicated that these data meet the SEM thresholds [53]. An MGA was performed to examine subgroup differences based on gender, age, marital status, experience, and department to confirm that observed differences reflected true variation among subgroups rather than artifacts.

## 5. Results

The general characteristics of the respondents included gender, marital status, age, education level, nationality, specialization, working experience, and department in which the respondent worked. These data are summarized in Table 1 according to frequencies and percentages. In total, 312 questionnaires were analyzed.

From the demographic summary, 61.5% were male and 38.5% were female, indicating that the Saudi public healthcare sector is mainly dominated by males. In terms of marital status, 66.3% of the participants were married, indicating that the majority of professionals were married. Furthermore, the distribution of the age of participants was as follows: 20–25 years, 4.8%; 26–30 years, 30.8%; 31–35 years, 35.6%; between 36 and 40 years, 22.1%; and above 40 years, 6.7%. The age range of most participants was 26–35 years. The educational levels were as follows: 20.2% had diplomas, 57.7% had bachelor’s degrees, 9.6% had master’s degrees, and 12.5% had doctoral degrees. Most graduates held a bachelor’s degree. The nationalities of the participants were as follows: 53.8% Saudi, 18.3% Egyptian, 1% Sudanese, 3.8% Indian, 21.2% Filipino, and 1.9% from other countries. Thus, most participants were Saudi citizens. Moreover, specializations were distributed as follows: 31.6% in cardiology, 53.8% in general healthcare and nursing, 7.7% in medical imaging and diagnostics, 4.8% in technology and engineering, and 1.9% in other fields. Most participants worked in general healthcare and nursing. Regarding work experience, 30.8% reported having up to 5 years of experience. Additionally, 51.9% had 6–10 years of experience, 14.4% had 11–15 years, 1.9% had 16–20 years, and 1% had more than 20 years of experience. Most respondents had 6–10 to years of service. The departmental distribution was as follows: 44.2% in cardiology, 4.8% in emergency and critical care, and 7.7% in medical imaging and diagnostics. Additionally, 30.8% were in general and medical services, 5.8% in nursing and medical support, and 6.7% in educational and technical support. The cardiology department had the highest level of representation. The departmental composition of the sample reflected staffing patterns within the Hail Health Cluster. It also resulted in a higher proportion of clinical employees, which may have influenced the representativeness of findings across occupational groups.

**Figure 3:**
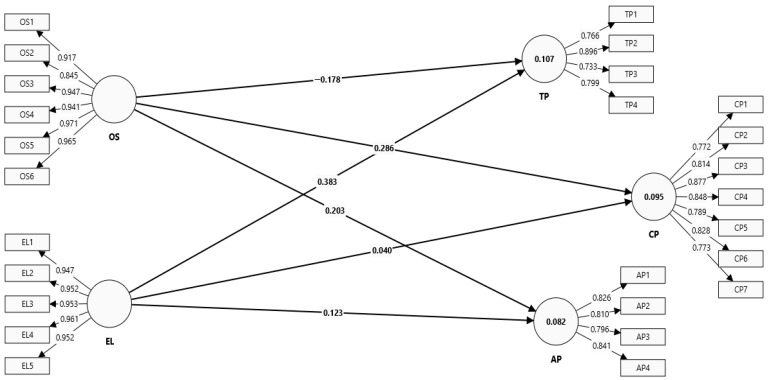
Measurement model.

Figure 3 shows the measurement model used in this study. This includes the exogenous constructs of EL and OS, along with the endogenous constructs of TP, CP, and AP. These are the dimensions of EP for the healthcare sectors in SA. As reflected in Figure 2, the indicators have a significant relationship with each other when their constructs are measured. According to Henseler et al. [54], an indicator with a standardized factor loading value of 0.70 or greater represents a significant indicator when measuring its construct. As the indicators indicated in Figure 2 meet the threshold of 0.70 or greater, all the constructs are significant when measured by their indicators.

Table 2 presents the results for factor loadings (FLs), variance inflation factors (VIFs), Cronbach alpha (CA), composite reliability (CR), and average variance extracted (AVE). These measures were used to assess the construct validity and reliability. According to the standard threshold values, a factor loading of 0.70 or above and a VIF below 5 indicate validity. Additionally, CA and CR should be 0.70 or above and AVE should be 0.50 or above to confirm the constructs’ reliability [55]. Table 2 shows that all constructs had FL, CA, and CR values greater than 0.70. The AVE value was above 0.50, and VIF was below 5.

Hair, Hult, Ringle, Sarstedt, Danks, and Ray [55] argued that discriminant validity is established when factors are significantly related to their relevant constructs rather than other constructs. The HTMT ratio and Fornell–Larcker criterion were used in this study. These are important methods for assessing discriminant validity as supported by most of the studies [56,57,58,59].

Table 3 reports the estimated HTMT ratios. These ratios should be less than 0.90 between correlated constructs to meet the criteria under this method [55]. The estimated values of HTMT ratios are less than the maximum threshold of 0.90, confirming the validity and reliability under this method.

Table 4 lists the $\sqrt{AVE}$ values for each construct representing the Fornell–Larcker criterion. To meet the threshold requirement using this method, the $\sqrt{AVE}$ of each construct must be >its correlational values with other constructs [55]. The estimated values of $\sqrt{AVE}$ (shown in bold) are >their correlational values. Therefore, the requirements of validity and reliability using this method are also confirmed.

Table 5 reports the model fit indices for the saturated model as well as the estimated model. To establish a good fit, an SRMR value < 0.08 and an NFI close to 0.90 are recommended [55]. Furthermore, the values for SRMR, d_ULS, d_G, Chi-square, and NFI in the saturated model should be close to those in the estimated model to ensure model accuracy and consistency [55]. All the SRMR and NFI values meet the threshold, and also, there is less variation between the saturated and estimated models; therefore, the model has good fit.

Although the model fit indices suggest a satisfactory fit, it is important to consider the limitations of the model. First, model fit statistics, such as SRMR and NFI, while informative, do not capture all aspects of model performance. For instance, these indices may not fully capture potential misspecifications in the model. They may also have overlooked omitted variables that could influence the results [60]. Additionally, fit indices, such as Chi-square, can be sensitive to sample size. This sensitivity may affect the interpretation of the goodness of fit of the model, particularly for large datasets [61]. Finally, while the close alignment between the saturated and estimated models is a positive indicator, underlying complexities in the data may still exist. The current model may not fully capture these complexities [62].

Table 6 shows the explanatory power of the model based on the *R*^2^, *Q*^2^, and *F*^2^ values. The significant values of these indices show that the model has a high explanatory level. *R*^2^ is the variance of the endogenous variable explained by the exogenous variables [63].

There are four significance levels for *R*^2^. Below 0.02 indicates no variation, 0.02–0.129 indicates weak variation, and 0.13–0.259 indicates moderate variation. A variation from 0.26 to 0.349 indicates significant variation, while values higher than 0.35 indicate highly significant variation [63]. As shown in Table 6, *R*^2^ explains the moderate-to-weak variation in the endogenous variables by EP. The *R*^2^ values are 0.155 for overall EP, 0.107 for TP, 0.095 for CP, and 0.082 for AP.

From a practical standpoint, these moderate *R*^2^ values highlight the importance of recognizing that factors beyond EL and OS may influence EP. Also, for the performance variables of the healthcare organizations, we should look beyond the basics: organizational culture, employee motivation, external factors, and job satisfaction. The leaders should adopt a holistic approach towards improving EP, including training, employee engagement, and resource optimization. While leadership does matter, it is not the only factor affecting performance. The *R^2^* values are low, which does not reduce the model’s contribution in any way [64]. Instead, this highlights that EP is a multidimensional construct. It is shaped by diverse personal, organizational, and cultural factors that extend beyond leadership and support [65].

The modest *R*^2^ values indicate that the model did not capture all the variance of EP and its subcategories due, for example, to relevant missing predictors or unconsidered latent variables [55]. Additionally, nonlinear relationships and measurement errors may obscure true associations [66]. Moreover, performance in healthcare organizations is strongly influenced by contextual factors such as patient load, inter-professional collaboration, policy frameworks, and intrinsic employee motivation [67]. These factors were not explicitly modeled. Accounting for such omissions helps to make sense of the less-than-perfect *R*^2^. To try improving *R*^2^, we should add these variables and look at nonlinear interactions. Improved measurements or larger samples might also enhance model fit and explanatory power [55].

*Q*^2^ in Table 6 shows the amount of predictive relevance that the exogenous variables exert on the endogenous variables. *Q*^2^ = 0 denotes no predictive relevance; *Q*^2^ = 0.01–0.25 means low predictive relevance; *Q*^2^ = 0.26–0.50 indicates moderate predictive relevance; and *Q*^2^ > 0.50 means high predictive relevance [63]. In this study, from Table 6, *Q*^2^ values estimated for the overall EP and for TP stand at 0.163. This signifies that the endogenous variables have low predictive relevance. This finding reflects the inherent complexity of performance, which cannot be easily predicted with a limited set of predictors. At the same time, it underscores the unique contribution of EL and OS. These variables explain variance despite the influence of many uncontrollable contextual factors.

Similarly, *F*^2^ describes the magnitude of the effect of one exogenous variable on another. The effect size can be classified as follows: *F*^2^ < 0.02 reflects no effect, *F*^2^ from 0.02 to 0.149 signifies a small effect, *F*^2^ ranging from 0.15 to 0.349 signifies a medium effect, and *F*^2^ ≥ 0.35 signifies a large effect [63]. The estimated *F*^2^ values for the overall EP as an endogenous variable were 0.387 for EL, 0.368 for OS, and 0.367 for the moderation of OS, all with large effect sizes. For TP, the *F*^2^ values were 0.120 for EL, 0.258 for OS, and 0.134 for the moderation of OS, reflecting medium effect sizes. For CP, the *F*^2^ values were 0.127 for EL, 0.166 for OS, and 0.125 for the moderation of OS, suggesting that all these effects fell into small-to-medium effect sizes. Finally, for AP, the *F^2^* values were 0.119 for EL, 0.233 for OS, and 0.134 for the moderation of OS, indicating small-to-medium effect sizes. This pattern shows that the model explains only a modest proportion of variance, as reflected in the low *R*^2^ value. Nevertheless, the substantive effect sizes of EL and OS remain meaningful, reinforcing their theoretical and practical importance.

**Figure 4:**
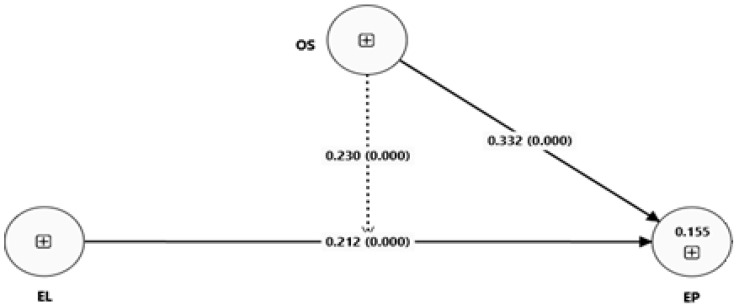
SEM model 1.

As shown in Figure 4, EL has a positive impact on EP. Moreover, OS has a positive effect on EP. Furthermore, OS strengthens the positive impact of EL on EP.

**Figure 5:**
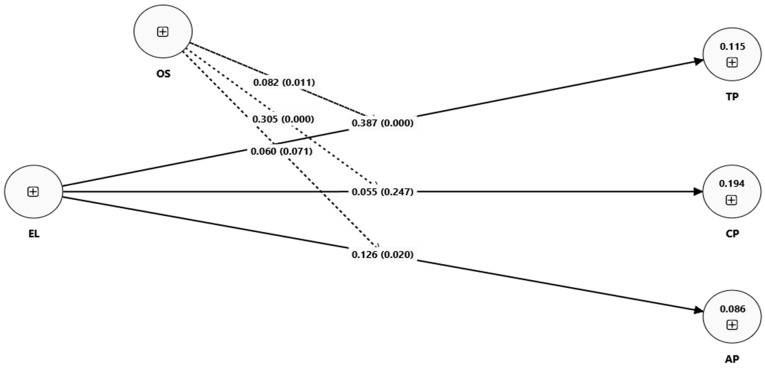
SEM model 2.

Figure 5 illustrates the moderating effect of OS for the impact of EL on TP, CP, and AP. It shows that OS strengthens the positive impacts of EL across all three dimensions of EP (TP, CP, and AP). Table 7 provides insights for more detailed analysis of these moderating effects and hypothesis testing.

Table 7 reports the coefficients and *p*-values related to direct as well as moderating impacts using the SEM approach of Smart PLS 4. The estimated results indicate a strong positive impact of EL on EP ($\beta_{1}$ = 0.212 and *p* = 0.000). Therefore, they support the first hypothesis. Additionally, the estimated results indicate a strong positive impact of EL on TP ($\beta_{1a}$ = 0.387 and p = 0.000). This supports the “H_1a_” hypothesis. Moreover, the estimated findings indicate a positive but insignificant impact of EL on CP ($\beta_{1b}$ = 0.055 and p = 0.247). This does not support the “H_1b_” hypothesis. Furthermore, the estimated findings indicate a positive and significant impact of EL on AP ($\beta_{1c}$ = 0.126 and p = 0.020). This supports the “H_1c_” hypothesis. Moreover, the estimated results indicate a strong positive impact of OS on EP ($\beta_{2}$ = 0.212 and p = 0.000). Thus, the second hypothesis is supported.

The estimated findings indicate a positive impact of OS in relation to EL and EP ($\beta_{3}$ = 0.230 and p = 0.000). Thus, the third hypothesis is supported. Similarly, the estimated findings indicate a positive and significant moderating impact of OS in relation to EL and TP ($\beta_{3a}$ = 0.082 and p = 0.011). This supports the “H_3a_” hypothesis. Furthermore, the estimated findings indicate a positive and significant moderating impact of OS in relation to EL on CP ($\beta_{3b}$ = 0.305 and p = 0.000). This supports the “H_3b_” hypothesis. Finally, the estimated findings indicate a positive and weakly significant moderating impact of OS in relation to EL on AP ($\beta_{3c}$ = 0.060 and p = 0.071). This supports the third “H_3c_” hypothesis.

The comparative analysis in Table 8 examines how EL and OS influence EP across demographic and organizational groups. These groups include gender (male, female), age (young, old), marital status (single, married), experience (junior, senior), and department (clinical, support). It is important to note that in terms of age, 20–35 years old is classified as young, while above 35 is older. Similarly, the departmental categories such as clinical staff include those personnel directly involved in patient care such as physicians, nurses, and allied health professionals. However, the support staff include personnel who do not provide direct clinical services such as operational, administrative, and technical staff. EL was highly effective for younger employees (β = 0.671 and p < 0.001). It was also significantly more effective for males compared to females (β = 0.210 and p = 0.048). Single employees benefitted more from EL than married employees (β = 0.492 and p = 0.031). Junior employees responded more positively than senior employees (β = 0.389 and p = 0.010). Departmental differences were not significant, suggesting consistent effects across clinical and support staff (β = −0.207 and p = 0.411). OS demonstrated significant differences. It was less effective for older employees compared to younger ones (β = −0.504 and p = 0.025). OS was highly effective for clinical departments compared to support departments (β = 1.207 and p < 0.001). Gender, marital status, and experience did not show significant differences in terms of the direct relationship between OS and performance. The moderating role of OS showed further variation. It significantly reduced the leadership-to-performance link among males (β = −0.295 and p = 0.015). It highly reduced the effect for senior employees compared to juniors (β = −0.378 and p = 0.002). Conversely, OS highly strengthened the relationship among clinical staff compared to support staff (β = 0.438 and p < 0.001). Marital status differences were weakly significant, with single employees showing slightly stronger moderation than married employees (β = 0.001 and p = 0.091). These results highlight systematic group variations, underscoring the importance of context-specific leadership and support strategies.

## 6. Discussion

This research explored how EL affects EP in the SA health sector and further investigated how OS can moderate this relationship. The results supported the hypotheses and showed that EL significantly affected EP in a manner that was consistent with prior research [26,68,69,70]. In line with Islamic principles that emphasize justice and fairness, EL serves as a moral compass for employees [71]. Ethical leadership molds staff behavior through integrity and also by aligning employee goals to organizational objectives, hence improving performance. This aligns with the cultural expectations of SA, where leadership is viewed not only as an administration but a religious and social obligation [72]. Such norms enhance the credibility of ethical leaders and extend their influence on EP more than in secular contexts, which indicates that EL theory is not culturally neutral. Its effectiveness will continue to vary and be shaped by religious, moral, and collectivist values dominating non-Western work settings [73]. The findings extend existing EL theory by placing it in the SA context, where leadership legitimacy derives both from ethical/religious alignment and from managerial authority.

This research shows that EL significantly enhances the breakdown of EP into TP, CP, and AP, thus stating that ethical leaders assist employees in managing change effectively within dynamic healthcare; adaptation remains important. The effect of EL on CP is positive yet not significant statistically, which contrasts with much of the literature on EL reporting strong links between EL and CP. A closer examination indicates that Saudi public healthcare organizations are highly formalized and hierarchical [74]. This structure leaves little room for discretionary or extra-role behaviors—those that are at the very core of CP. In a compliance-driven system, authority, protocols, and formal accountability prevail, whereby employees are not likely, or perhaps even not encouraged, to exhibit voluntary, citizenship-like actions. This goes against earlier findings where better EL–CP effects have been found [26,70]. The weak EL–CP link here does not mean EL failed; it shows how organizational hierarchy and cultural norms favor task completion and role adherence over discretionary contributions [75]. This leads employees to focus on the core task-related and adaptive behaviors that are directly rewarded or monitored, while voluntary extra-role contributions remain limited. This explanation extends the EL theory by highlighting its contingency: the impact of EL on CP is contingent on organizational structural rigidity and cultural hierarchy [76]. This divergence suggests that the theory of EL should be further refined for rigid hierarchical non-Western organizations, where institutional structures more strongly constrain contextual and extra-role behaviors than individual leader–employee dynamics.

OS was also found to significantly improve EP, supporting past studies that emphasized its role in performance and well-being [77,78]. In the high-stress setting of Saudi public healthcare, OS provided fundamental resources, emotional support, and enabling conditions to sustain peak performance. As opposed to some earlier studies, where OS mainly enhanced well-being, in Saudi public healthcare, its effect on performance was more evident. Here, “visibility” refers to the heightened awareness of employees regarding supervisory recognition and fairness. Furthermore, it also includes access to support resources in public-sector institutions where hierarchical structures and centralized decisions raise the stakes regarding supportive leadership. This is particularly so for clinical staff under high workload pressures with limited alternative coping options. This difference arises from extreme demands on clinical staff and the scarcity of alternative coping mechanisms. The findings advance the theoretical understanding by demonstrating the critical role of OS. In Western studies, support is often portrayed primarily as a facilitator of well-being [79]. In non-Western, resource-constrained environments, however, it emerges as a structural necessity for enabling ethical leadership to translate into performance.

The MGA results also indicated subgroup differences. EL affected younger, male, single, and junior employees more strongly, with reduced impact on senior or married employees. OS had a greater influence on clinical staff, where workload is high, than on support staff. The findings related to demographic differences are vital for interpreting CP. For example, CP was not directly influenced by EL. However, the differences across demographic groups suggest that CP may depend more heavily on these demographics based on their organizational and contextual settings. These might include hierarchy, culture, job demands, and role expectations rather than leadership behaviors alone. Specifically, OS had the highest moderating effect on clinical and junior staff and a little less of an effect on seniors and males. This depicts how demographic and organizational contexts shape certain outcomes well. For instance, juniors and clinical employees are more dependent on ethical guidance and organizational support because of higher uncertainty and pressure [80]. In contrast, senior or support staff rely more on professional experience or established routines [81]. These subgroup differences extend EL theory by highlighting its contingent nature. The influence of EL depends not only on leader behavior but also on employees’ demographic and role characteristics [29,38,72]. In summary, these results emphasize that both EL and OS operate in a context-dependent manner. Furthermore, younger employees and clinical staff may be especially responsive to EL practices. This may be due to higher uncertainty, role ambiguity, and frontline pressures. The results show that CP remained unaffected by EL, which suggests that CP relies more on organizational factors such as hierarchy, culture, and workload than on leadership ethics alone [76].

Furthermore, OS moderates the EL–EP link affecting overall EP as well as TP, CP, and AP. With high OS, EL’s positive effect strengthens, showing that leadership alone cannot bear fruit without support structures and psychologically supportive conditions [82]. Conversely, when OS is weak, even strong ethical leadership is less effective, which contrasts with less demanding contexts where leadership alone may suffice. In Saudi public healthcare, work pressures are extreme, and hence OS becomes imperative to translate leadership values into performance gains. It is in supportive settings that ethical leaders inspire employees more effectively [23]. They encourage core task engagement, discretionary behaviors, and adaptive responses to change within the workplace. This study deepened the theoretical application of EL to demonstrate how the outcomes of EL depend on institutional resources and contextual realities, hence explaining why CP might remain unaffected in rigid, compliance-driven systems.

This study contributes practical, empirical, and methodological dimensions. The practical contribution lies in providing evidence-based guidance for leadership development and workforce planning in Saudi public hospitals. Here hierarchical structures and heavy workloads make EL and OS particularly important. It identifies groups, including clinical and younger staff, who may benefit most from targeted interventions aimed at enhancing leadership and support. Therefore, it informs policy and management reforms. On the empirical level, it extends the literature by simultaneously investigating EL and OS in a Middle Eastern public healthcare setting. Until now, this has been understudied. Methodologically, demographic segmentation and multigroup analysis provide greater nuance into how the relationships between leadership and performance vary between subgroups, offering a context-sensitive, analytically differentiated perspective.

## 7. Conclusions

This study investigates the effect of EL on EP, moderated by OS, in SA public healthcare. It finds that both EL and OS have a positive and significant effect on EP, and further, that OS enhances EL’s effect on EP. MGA leads to subgroup differences by gender, age, marital status, experience, and department, which suggests that EL and OS’s effects vary depending on employee and organizational traits.

These findings support SA’s Vision 2030 for transforming healthcare through the enhancement of EL and improvement of EP, thereby strengthening OS. This study, in brief, underlines the fundamental role of EL to help increase the quality of care towards a patient-centric, efficient system. Positive impacts of EL and OS on EP represent a milestone, so far, toward reaching these goals and portend a healthier, more sustainable future for the population.

Beyond policy, this study offers theoretical value in explaining why EL and OS diverge from findings in other contexts. Saudi public healthcare is highly hierarchical and role-focused, with strong cultural and religious norms shaping responses to leadership and OS. Notably, EL did not significantly influence CP, which runs somewhat against much of the literature findings. This suggests that rigid hierarchies and compliance-focused systems limit discretionary, extra-role behaviors, and hence CP does not follow automatically from EL in this setting. This work extends theory by demonstrating organizational structure and cultural constraints as critical boundary conditions, better clarifying the EL–EP link and the necessity for theory to account for setting-specific dynamics.

### 7.1. Theoretical Implications

This research makes a new contribution to EL theory, especially in Saudi Arabia’s public healthcare sector, showing how EL influences performance. EL—through leaders demonstrating integrity and ethics—enhances performance in all roles, from doctors and nurses to administrative staff, confirming the universal influence of EL on high performance. Also, it extends the theory of EL by demonstrating that its influence is moderated by socio-cultural and institutional norms outside of the West: religious values, collectivism, and hierarchical authority enhance the credibility of ethical leaders and amplify performance, extending EL beyond Western assumptions.

Organization support emerges as an important contextual factor that positively influences EP and strengthens the positive spillover effect of EL. OS as a moderator opens up new avenues of research and invites consideration of other contextual factors interacting with EL to influence performance. In the demanding, hierarchical context of Saudi public healthcare, OS is not a choice but an imperative if EL is to yield outcomes.

These findings explain why CP, considered within the context of Saudi public healthcare, is weaker compared to previous studies, and theoretically underline the importance of hierarchy and role rigidity as boundary conditions. In cultures with constrained discretionary behavior, EL may influence TP and AP more than CP, so boundaries like these should be included in EL theory since such role rigidity, compliance structures, and cultural constraints alter EL effects across performance dimensions.

The MGA results further extend the theory by displaying subgroup variations: demographic and organizational contingencies shape the influence of leadership and support on performance and point to the need for subgroup dynamics to be integrated into leadership models. EL exhibits a significantly stronger influence on junior, clinical, and less experienced staff than on either senior or support staff, thereby identifying demographic contingencies that condition EL’s effects and informing future theory building considering workforce heterogeneity.

Overall, this study broadens the theory of EL by showing general effects on employees and conditional effects shaped by cultural, structural, and subgroup characteristics. Importantly, it situates EL in a non-Western healthcare-specific context and exemplifies that cultural norms, institutional structures, and subgroup dynamics all serve as boundary conditions in shaping the mechanisms through which EL’s influence operates and in shaping the strength of such influence.

### 7.2. Practical Implications

The present study provides important lessons for healthcare leaders in SA on both EL and OS for enhancing EP. Ethically sound, transparent, and integrity-driven leadership—doctors, nurses, and admins—can uplift team performance. SA public healthcare units are strongly encouraged to focus on the development of leadership programs, incorporation of ethics into performance measures, and establishment of a trusting, efficient work environment.

OS directly enhances EP and strengthens EL’s impact, ensuring a supportive workplace in which staff are valued, receive regular feedback, and have their needs met with resources. Treating OS as a moderator maximizes the benefits of EL. Strategies should be tailored to subgroups; for example, junior staff require EL with mentorship in a structured environment, while clinical staff benefit from OS initiatives. Senior staff respond to recognition and appreciation. This is why leadership and support differ from those in other countries, especially in hierarchical and high-pressure settings. Targeted support to vulnerable groups is critical.

Allocate resources to align EL and OS: provide appropriate staffing to prevent burnout, ongoing professional development, and adequate financial and technological support for ethical practice and well-being. Investing in training leadership, ethics-based decision-making, and supportive infrastructure can translate EL and OS into real performance gains.

The combination of EL and OS holds promise for a more motivated, committed, and high-performing workforce, which will improve patient care and organizational effectiveness in SA’s unique context. Managers will need to adapt these EL and OS strategies for Saudi public healthcare, where many strategies that work in less hierarchical or Western systems necessarily require adjustment.

### 7.3. Limitations and Future Research Directions

While informative, the narrow focus of this study on the health sector in SA limits generalizability to other sectors or cultural contexts. As the samples are drawn from only one region, this limits its applicability to the wider Saudi healthcare system. Results might also not capture the dynamics of different cultural or organizational settings, calling for future research across varied regions and sectors. The sample only involved public hospitals from one regional cluster. Therefore, the findings cannot be generalized to private-sector institutions or other Saudi regions due to different leadership, staffing, or resources. Similar studies conducted in other countries and industries can help to increase generalizability. Future cross-country comparisons would yield stronger evidence of the universality or context-specificity of EL and OS and allow testing of the proposed contextual explanations, such as cultural–religious norms, hierarchical structures, or subgroup dynamics, which might explain divergences from prior findings.

A second limitation concerns the departmental composition of the sample. While participants were drawn from multiple specialties, the distribution was somewhat skewed toward cardiology and general healthcare units. Clinical units (emergency, inpatient wards, and outpatient clinics) were more represented compared with administrative and support departments. This may limit generalizability because support staff’s experiences and perceptions may not be fully captured. Thus, OS, perceptions about leadership, and performance outcomes should be considered with caution, and further studies should try to sample more departments. Experiences and performance dynamics vary across departments, such as emergency care, medical imaging, support services. Future research should make sure that specialty representation is balanced in order to capture diversity in healthcare contexts and make cross-departmental comparisons to determine if the effects of EL and OS are consistent or vary by clinical function.

The cross-sectional design of this study limits the testing for causation between EL and EP. Longitudinal studies are needed to collect data at multiple time points, over one to three years, to assess how changes in EL behavior affect EP over time. Including moderators such as cultural values and leadership styles—for example, Hofstede’s dimensions, including power distance and collectivism—will help to better articulate when EL and OS influence performance and why effects differ across settings. Examining mediators such as job satisfaction, trust, and psychological safety will explain how EL and OS take shape in performance and illuminate the processes underlying such links.

The low R^2^ values of EP and its subdimensions prove that though EL and OS do matter, not all the variance is explained. Self-reporting might have invited social desirability bias as well. There are certain other factors, like job satisfaction, organizational culture, motivation, and external environment, which have not been measured but must be affecting the outcomes. Such variables must be included in future work for an improved model.

SA public healthcare organizations are highly hierarchical, which may bias responses due to power distance or fear of repercussions. Future studies should address hierarchical effects via mixed methods or stronger confidentiality assurances.

Low Q^2^ indicates that stronger modeling is required. Nonlinear relationships and latent constructs, such as psychological safety and resilience, are important areas for future research. For multilevel modeling, separating the individual and organizational factors might be considered. The use of longitudinal or mixed-methods designs will enhance causal inference and might offer valuable insights into how such dynamic relationships can be modeled. These combined approaches will provide a more complete understanding of EL, OS, and EP in a health setting. By doing so, subsequent studies can capture the multidimensional nature of performance more effectively.

## Figures and Tables

**Table 1:** Respondent’s characteristics.

Characteristics	Categories	Frequency	Percentage
Gender			
	Male	192	61.5%
	Female	120	38.5%
Marital Status			
	Single	105	33.7%
	Married	207	66.3%
Age (in years)			
	20 to 25	15	4.8%
	26 to 30	96	30.8%
	31 to 35	111	35.6%
	36 to 40	69	22.1%
	>40	21	6.7%
Education (by degree)			
	Diploma	63	20.2%
	Bachelor	180	57.7%
	Master	30	9.6%
	Doctorate	39	12.5%
Nationality			
	Saudi	168	53.8%
	Egyptian	57	18.3%
	Sudanese	3	1.0%
	Indian	12	3.8%
	Filipino	66	21.2%
	Other, if any……	6	1.9%
Specialization			
	Cardiology	99	31.7%
	General Healthcare and Nursing	168	53.8%
	Medical Imaging and Diagnostics	24	7.7%
	Technology and Engineering	15	4.8%
	Others	6	1.9%
Experience (in years)			
	≤5	96	30.8%
	6–10	162	51.9%
	11–15	45	14.4%
	16–20	6	1.9%
	>20	3	1.0%
Department			
	Cardiology	138	44.2%
	Emergency and Critical Care	15	4.8%
	Medical Imaging and Diagnostics	24	7.7%
	General and Medical Services	96	30.8%
	Nursing and Medical Support	18	5.8%
	Educational and Technical Support	21	6.7%

**Table 2:** Convergent validity.

Constructs and Their Factors	FL	VIF	CA	CR (rho_a)	CR (rho_c)	AVE
Adaptive Performance			0.838	0.854	0.890	0.670
AP1	0.826	2.189				
AP2	0.810	2.052				
AP3	0.796	1.710				
AP4	0.841	1.751				
Contextual Performance			0.919	0.987	0.933	0.665
CP1	0.772	1.999				
CP2	0.814	2.775				
CP3	0.877	2.566				
CP4	0.848	2.905				
CP5	0.789	2.677				
CP6	0.828	3.649				
CP7	0.773	2.649				
Ethical Leadership			0.975	0.999	0.980	0.908
EL1	0.947	2.581				
EL2	0.952	1.764				
EL3	0.953	2.088				
EL4	0.961	3.200				
EL5	0.952	2.133				
Organizational Support			0.969	0.977	0.975	0.869
OS1	0.917	2.374				
OS2	0.845	2.982				
OS3	0.947	3.933				
OS4	0.941	1.097				
OS5	0.971	1.311				
OS6	0.965	1.742				
Task Performance			0.813	0.830	0.877	0.641
TP1	0.766	1.589				
TP2	0.896	2.442				
TP3	0.733	1.643				
TP4	0.799	1.933				

**Table 3:** HTMT ratio.

	AP	CP	EL	OS	TP
AP					
CP	0.415				
EL	0.249	0.168			
OS	0.279	0.273	0.532		
TP	0.605	0.255	0.307	0.093	

**Table 4:** Fornell–Larcker criterion.

	AP	CP	EL	OS	TP
AP	0.819				
CP	0.375	0.815			
EL	0.228	0.189	0.953		
OS	0.267	0.307	0.521	0.932	
TP	0.488	0.220	0.290	0.022	0.801

**Table 5:** Model fit.

Indices	Saturated Model	Estimated Model
SRMR	0.072	0.072
d_ULS	1.836	1.796
d_G	1.954	1.943
Chi-square	5066.144	5011.183
NFI	0.843	0.831

**Table 6:** Model explanatory power.

Constructs	R^2^	Q^2^	F^2^			
EP	0.155	0.163	EP	TP	CP	AP
AP	0.082	0.076	-	-	-	-
CP	0.095	0.087	-	-	-	-
TP	0.107	0.101	-	-	-	-
EL	-	-	0.387	0.120	0.127	0.119
OS	-	-	0.368	0.258	0.166	0.233
OS × EL → EP	-	-	0.367	0.134	0.125	0.134

**Table 7:** SEM estimates (hypothesis testing).

Relationships	Coefficients	*p*-Values	Decision
Direct Impacts			
EL → EP	0.212	0.000	Supported H_1_
OS → EP	0.332	0.000	Supported H_2_
EL → TP	0.387	0.000	Supported H_1a_
EL → CP	0.055	0.247	Unsupported H_1b_
EL → AP	0.126	0.020	Supported H_1c_
Moderating Impacts			
OS × EL → EP	0.230	0.000	Supported H_3_
OS × EL → TP	0.082	0.011	Supported H_3a_
OS × EL → CP	0.305	0.000	Supported H_3b_
OS × EL → AP	0.060	0.071	Supported H_3c_

**Table 8:** A comparative group analysis.

Differences						
Paths	Male–Female	Young–Old	Single–Married	Junior–Senior	Clinical–Support	Decision
EL → EP	0.21 (0.048)	0.671 (0.000)	0.492 (0.031)	0.389 (0.010)	−0.207 (0.411)	Supported H_4a_
OS → EP	−0.12 (0.413)	−0.504 (0.025)	−0.315 (0.468)	−0.473 (0.172)	1.207 (0.000)	Supported H_4b_ for age and department type
OS × EL → EP	−0.295 (0.015)	−0.041 (0.721)	0.001 (0.091)	−0.378 (0.002)	0.438 (0.000)	Supported H_4c_ for all except gender and experience

Note: *p*-values in parentheses.

## Data Availability

Data supporting the findings of this study are available from the corresponding author upon reasonable request.
